# Experimental Study on Hybrid Additive and Subtractive Manufacturing Processes for Improving Surface Quality

**DOI:** 10.3390/ma18133136

**Published:** 2025-07-02

**Authors:** Monika Jabłońska

**Affiliations:** Faculty of Mechanical and Electrical Engineering, Polish Naval Academy, Jana Śmidowicza 69, 81-127 Gdynia, Poland; m.jablonska@amw.gdynia.pl

**Keywords:** additive manufacturing, hybrid techniques, surface roughness, milling, surface quality, PETG, surface topography, microstructure

## Abstract

Hybrid machining has considerable potential for industrial applications. The process allows the limitations of additive manufacturing to be reduced and high-precision components to be produced. This article discusses tests determining the impact of 3D printing parameters, machining parameters, and selected milling tools on achieving defined surface roughness values in parts made of PETG (polyethylene terephthalate glycol). Perpendicular-shaped samples were printed by fused deposition modelling (FDM) using variable layer heights of 0.1 mm and 0.2 mm and variable feed rates of 90, 100, 110, and 120 mm/s. Surface roughness values, topography, and Abbott–Firestone curves were determined using a Keyence VR-6000 profilometer. Straight grooves were machined in the test samples using a DMG MORI CMX 600V milling machine with a rotary burr, single-edge spiral burr cutter and spiral endmill. The microstructure was examined using a Motic inverted microscope. The surface roughness parameters of the grooves were investigated. The results confirmed that the use of hybrid machining (with a printed layer height *L_h_* = 0.1 mm, *V_feed_* = 120 mm/s, and a cutter–rotary burr) allows for lower surface roughness parameters, i.e., Ra = 1.54 μm. The relationships developed between printing, cutting, and milling tool parameters can be employed to predict the roughness parameters of filaments with similar characteristics.

## 1. Introduction

Hybrid machining is the combination of two material processing methods, e.g., machining and additive machining, into a single manufacturing process. Current trends in manufacturing, particularly in the micro- and nano-scale areas, involve merging complementary technologies to overcome the limitations of individual processes. Hybrid processes can integrate various machine methods, such as mechanical machining and electrochemical (ECM), mechanical machining and electric discharge machining (EDM), mechanical machining and laser cutting, turn–milling, laser cladding and mechanical machining, arc welding and mechanical machining, additive/subtractive and transformative manufacturing processes, additive and subtractive processes, and many others. The aim of hybrid machining is to optimize process efficiency, increase manufacturing precision, improve surface quality, reduce tool wear, shorten production time, and lower tooling costs. Integrating several technologies into a multi-step process chain enables further improvements in manufacturing accuracy and efficiency, while reducing the technological and economic barriers typical of manufacturing. The design of the process chain in hybrid machining should take into account the required tolerances and surface finishes, the number of changeovers, station configurations, and the strategy for scheduling subsequent operations. It is worth noting that the use of hybrid machining is associated with the occurrence of so-called technological fingerprints, i.e., unintended process effects typical of the technology included in the chain. These fingerprints include, among others, changes in the microstructure of the material in the machining zone, the appearance of microporosity, burrs, deposits, and surface contaminants. The occurrence of such process effects can significantly affect subsequent operations and the quality parameters of the final product. Therefore, technological fingerprints must be identified and analyzed during the planning phase of the manufacturing process. Taking them into account in the design of the technological chain is crucial to ensure the repeatability and expected quality of the final product [1,2,3]. The synergy of combined processes allows for significant benefits to be achieved and is also in line with Industry 4.0 strategy [4,5]. Reduction of waste, reduction of power consumption, increased efficiency, digitisation, and use of new technologies are essential aspects of this strategy, which are reflected in hybrid machining [6].

The significance of hybrid techniques in the processing of materials is systematically increasing. The market related to 3D printing products and services is expected to grow by 17% per year, with an estimated value of around USD 12.6 billion in 2020 [7]. Such dynamic development of modern technologies and materials and the increasing demands for the quality of the final component are driving the search for solutions that guarantee low costs, short manufacturing times, fulfilment of quality requirements, and machining accuracy [8].

Incorporating machining into the additive process offers excellent potential for industrial use, as it overcomes the limitations of additive technology and obtains high-precision parts [9]. Combining these two technologies also enables production optimisation by leveraging the advantages of both processes while minimizing their disadvantages. In recent years, various hybrid techniques have been developed, such as hybrid layer manufacturing (HLM) [10,11], hybrid plasma deposition and milling (HPDM) [12], and 3D welding and milling [13].

The necessity of hybrid processing stems from the limitations of the manufacturing processes that compose it. Three-dimensional printing, despite its many advantages (e.g., the ability to produce complex geometries and efficiency in the use of building materials), has limitations, e.g., the need for finishing to improve surface quality after the printing stage or the inaccurate reproduction of component geometry [14,15,16]. Print parameter optimisation refers to identifying the optimal print parameters (e.g., layer height in a single pass, fill percentage, filament layer deposition speed, number of outer walls, work table temperature, and extruder temperature) that will enable the production of parts with the most accurate geometry and low roughness parameters. The selection of the right printing parameters will improve the mechanical strength of the parts. With the right choice of nozzle temperature comes smooth melting, better fusion of deposited layers of the filament, and an increase in the tensile strength and elastic modulus of the printed part [17]. Other benefits of optimisation include reduced shrinkage, increased process repeatability, and lower production costs [18].

Combining traditional production methods, such as milling, with rapid prototyping techniques can expand or create new production solutions that improve the geometric cooperation of components, accuracy, and damage repair [19]. The quality of the functional surface of cooperating elements, e.g., a machine shaft with a gear wheel, can be improved by finishing with milling keyways in both elements [20]. In this case, additive manufacturing generates the geometry, whereas machining is implemented to achieve the required dimensional tolerances and surface geometry parameters [21].

The machining stage is most often employed to remove support elements after printing or surface finishing. Removing support structures manually generates many issues, including limitations on the geometry of the printed part, as it is necessary to access the structures with your hand or a tool, leading to increased workload and longer production times. However, using different support methods during printing is the reason for this component’s different surface roughness values [22]. Implementing milling technology after the additive manufacturing stage enables the required surface quality, allowing defects to be eliminated and accurate dimensions to be obtained quickly [23]. Mention should also be made of the phenomenon of jaggies, or the build-up effect, which occurs during 3D printing [24,25]. Discontinuities in the layer structure, resulting from the infill used and the number and thickness of layers and shrinkage, can lead to incorrect geometry reproduction and large dimensional deviations [26]. Finish machining allows these errors to be eliminated.

The buy-to-fly ratio is the ratio of raw material to the finished part. Printed parts have a factor of almost 1, while fully milled parts can have a factor of up to 20 if 95% of the semi-finished part has been machined [27]. Hybrid technology makes it possible to design and manufacture parts individually, lowering the buy-to-fly ratio and reducing material waste.

It should be noted that the integration of 3D printing with modern CNC machining aligns with the principles of a circular economy. Sustainable production, recycling, and the possibility of reusing resources contribute to reducing waste generation while also aligning with the European Commission’s strategy to promote plastic waste management. The aim is to increase the reuse and recycling of plastics [28,29] to 55% by 2030. Sustainable production is the key term; it refers to manufacturing products in such a way as to minimize environmental impact and rationally manage natural resources.

Using a hybrid manufacturing process to produce the part also increases design flexibility and thus enables the production of customized components [30].

Integrating both techniques into a single workstation poses a design and technological challenge. The need to set print parameters for each component and to calibrate according to the geometry of the CNC part means that the integration of these techniques in industry is limited [31].

CNC machining has become increasingly indispensable in recent years. This is linked to reliability, efficiency and high-dimensional accuracy. Milling allows for a reduction in surface roughness and an improvement in functional properties. The tool (cutter) rotates around its axis during machining, and the workpiece moves along the selected path. Compared to the traditional milling process, when creating a machining strategy dedicated to machining a workpiece on a CNC machine tool, it is possible to set many cutting parameters. A processing operation involves several variables that control the process. These variables are, for example, feed rate *F*, milling depth *a_p_*, milling width *a_e_*, and spindle direction. The ability to optimize the tool path is also essential. Selectable milling methods include co-rotating or counterrotating, use of trochoidal machining, and an indication of how the cutter enters the material (inclined, helical or perpendicular, or tangential) [32,33,34]. By optimizing the input factors in the milling process, a reduction in the surface roughness parameter can be achieved, and the machining time can be reduced.

The effect of CNC milling parameters on surface roughness using PETG (polyethylene terephthalate glycol) material was discussed by El Mehtedi et al. [35,36]. The PETG material used in this process originates from FDM (fused deposition modelling), an additive manufacturing technology. According to the results, an increase in spindle speed, a lower feed rate, and a lower depth of cut improve surface finish. However, this increases the risk of burrs forming, which require an additional technological operation to remove. Milling operations enabled the correction of dimensional inaccuracies present in the part. ML Dezaki et al. [37] showed that the orientation of the print layer’s overlay influences the surface roughness value. Finish machining can improve surface quality. The material used in this research originates from FDM. Jabłońska M. et al. [38] discuss the use of hybrid technology that combines 3D printing and turning. Three-dimensional printing reduces production time and reduces waste, while turning compensates for imperfections and dimensional differences. The authors demonstrated that combining these two manufacturing processes offers greater versatility and expanded manufacturing capabilities.

The relationships between cutting parameters and the surface roughness of an FDM-printed part are complex and require further discussion. The inhomogeneity of the surface structure, the difficulty of obtaining the required roughness, and the limitations associated with the selection of cutting parameters for additive materials are challenges that concern hybrid machining [39]. There is a research gap in this area: a lack of information on how milling parameters affect a specific material, such as PETG, when used with particular 3D printing parameters in additive technology. Additionally, the tools used for the milling stage are also an important consideration. Tool manufacturers do not provide information on the machining parameters to use for a given polymer material (e.g., PETG) or the roughness that will be obtained. This is an unexplored area. There is also a lack of comparison between surface finishing techniques and materials used in additive technology [40]. Procedures are currently being developed to generate models for predicting surface roughness in a hybrid manufacturing process using neural networks [14]. However, to obtain more accurate models and predictions, the number of input parameters should be increased. For example, this could be achieved by including the printing parameters, which are covered in the studies described in this article. This would enable the database to be enlarged.

The study aims to illustrate how 3D printing and milling parameters and the milling tools selected influence the surface quality of 3D-printed parts. In particular, the study focuses on the printing process concerning layer height, filament deposition speed, and factors related to machining, including spindle speed, feed rate, and the three types of end mills used for machining. The surface roughness parameters chosen for the study are Ra and Rz. These indices provide essential information about surface topography characteristics. They are among the most commonly used parameters for evaluating surface quality after machining in manufacturing companies. Evaluating these parameters makes it possible to predict the sequence of operations necessary to achieve the required surface quality and optimize the production process. The results can be applied by technologists and designers in real production processes, thus strengthening the connection between science and industry [41,42].

The article is divided into the following sections. Section 1 provides a literature review and discusses the definition and role of hybrid techniques in the processing of materials. Section 2 describes the material, the object of the study, and the testing algorithm used. Section 3 covers the results and discussion. Section 4 addresses the future development of hybrid technology.

An essential aspect of this study is the use of an innovative hybrid PETG material technique. This study can be used to plan the hybrid machining process. Our multi-criteria analysis of selected milling tools highlights the advantages and identifies the risks associated with their use in machining additive manufacturing components.

## 2. Materials and Methods

### 2.1. Filament Material

Polyethylene terephthalate glycol (PETG) from Rosa (ROSA PLAST Sp. z o.o., Hipolitów, Poland) was used to produce the samples. This thermoplastic polymer is known for its low shrinkage, chemical resistance, high strength, lack of brittleness, excellent film adhesion, and ability to be recycled many times in thermomechanical processes without diminishing [43,44].

The selected physical properties of the PETG material were measured as follows:

- Density (1.29 g/cm^3^);
- Tensile modulus (2980 MPa);
- Tensile stress at yield (51 MPa);
- Izod impact strength (4.7 kJ/m^2^).

Another important factor in choosing a filament for part manufacturing was the ability to map the geometry of parts with high accuracy during printing. The PETG filament is characterized by good adhesion to the work surface, which translates into the dimensional accuracy and repeatability of the manufactured parts. The material has a low absorption coefficient of about 0.16%, which minimizes the risk of print quality deterioration in changing environmental conditions. PETG is also characterized by increased impact strength and resistance to permanent deformation, which is why it is recommended for manufacturing mechanical parts.

The filament, a 1.75 mm diameter monofilament, was wound onto a spool and dried in a drying oven at 60 °C for four hours before printing.

According to the filament manufacturer, the recommended printing parameters are extruder temperature *E_t_* = (220–250) °C and platform temperature *P_t_* = (60–80) °C.

To determine the optimum printing parameters, a series of calibration cubes with sides equal to 20 mm were printed. In subsequent trials, the work table and print head temperature were changed in 5 °C increments within the temperature range recommended by the filament manufacturer. These steps were repeated until the geometry was correctly reproduced and the assumed surface quality was achieved.

### 2.2. Object of the Study

**Figure 1:**
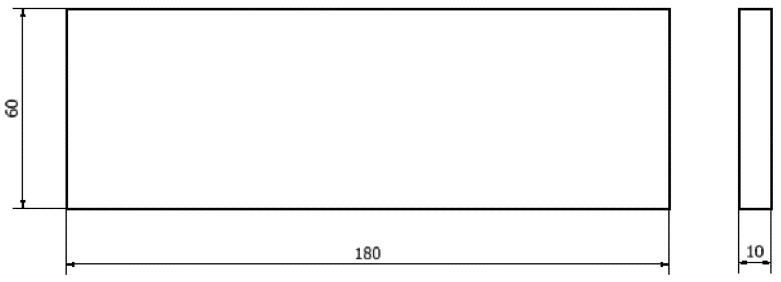
Model of the test specimen 180 × 60 × 10 mm.

The object of the study (Figure 1) was modelled using Autodesk Inventor Professional software (Version: Inventor 2025.1, Autodesk Inc., San Francisco, CA, USA). It was then converted into an STL file. When saving the file, a binary format, a surface deviation of 0.05%, a standard deviation of 1, and a maximum edge length of 1% were selected, and the transfer of internal mesh nodes was allowed. The model was afterwards transferred to Slicer Voxelizer Industry Software (Version: Voxelizer Industry 2.0.0, Zmorph S.A., Wrocław, Poland). The software enabled G-Code to be generated using the parameters in Table 1 for incremental FDM (fused deposition modelling) printing of 8 samples on a Zmorf i500 industrial printer (Zmorph S.A., Wrocław, Poland) in PETG material. The printer has a build chamber with a 460 × 300 × 500 mm workspace and is designed for rapid prototyping. The samples were printed individually and placed on the worktable closer to the centre of the printer table. The worktable and nozzle were cleaned before starting the printing process. The table’s surface was lubricated with adhesive to increase the adhesion of the first print layers to the working table. At the time of printing, the ambient temperature was 23 °C and the relative humidity in the room was maintained at 40%.

The variable FDM parameters used during printing were the height of the filament layer deposited in one pass, *L_h_* = (0.1; 0.2) mm, and the feed rate during which the filament layer was extruded, *V_feed_* = (90; 100; 110; 120) mm/s (Table 1). A printhead nozzle diameter of 0.4 mm was employed. According to data from the literature [45,46], the proportion of air voids between the print rasters and the porosity increase as the nozzle diameter increases.

The terms, definitions, and symbols of incremental manufacturing are standardized according to ISO/ASTM 52900 [47,48]. The terminology used in the current study is based on its provisions.

### 2.3. Testing Algorithm

The testing was divided into several stages and performed at the Naval Academy, Gdynia, Poland.

The experiment process used included the following operations:

1. Analysis of the surface topography of samples after the printing stage using a Keyence VR 6100 profilometer (KEYENCE CORPORATION, Osaka, Japan).
2. Milling operation using a CMX 600V Vertical Machining Centre (DMG, Pleszew, Poland).
3. Examination of the surface roughness of the specimen after milling using a Keyence VR 6100 profilometer (KEYENCE CORPORATION, Osaka, Japan).
4. Analysis of photographs of the microstructure of the groove surfaces of the samples using a Keyence VR 6100 profilometer (KEYENCE CORPORATION, Osaka, Japan) and Motic inverted microscope (Motic, Xiamen, China).

**Figure 2:**
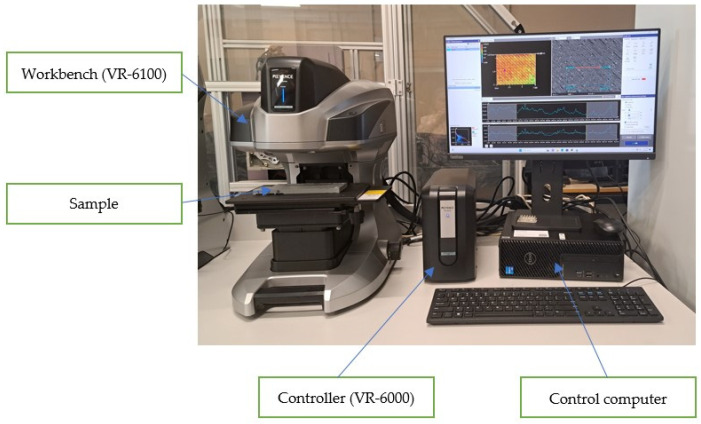
Surface topography measurement—Keyence VR-6100.

The first stage involved analysing the topography of the sample surfaces, as shown in Figure 2. Keyence’s VR-6100 optical 3D profilometer was used to study the topography. The profilometer uses advanced light-sectioning technology for dimensional measurement. The transmitter emits structured light onto the object’s surface, and the receiver records the reflected rays by analysing linear changes and curvatures caused by surface irregularities. The heights of individual points of the object were calculated based on this using the triangulation method. The optical 3D profilometer consists of the main device, that is, the scanner (VR-6100); a Keyence dedicated controller (VR-6000); and Keyence VR Studio software Version: VR-6000, KEYENCE CORPORATION, Osaka, Japan).

The technical data of the profilometer is defined in Table 2.

The surface topography was assessed quantitatively by analyzing 2D roughness parameters (*Ra*—arithmetic mean of the ordinates of the roughness profile and *Rz*—maximum height of the roughness profile), i.e., roughness profile lines, and qualitatively by assessing isometric views of the measured surfaces [41], i.e., line roughness measurements over a specified cross-sectional shape.

**Figure 3:**
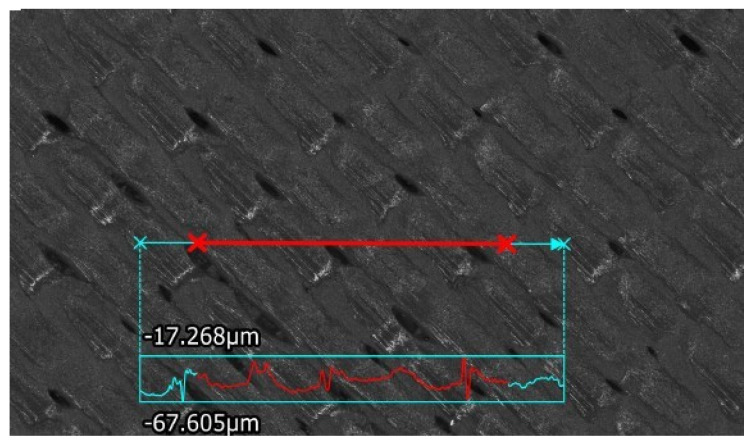
Specimen No. 3: the direction of the surface roughness measurement profile with respect to the material deposition tracks.

Measurement data were obtained by a high-magnification camera (high-resolution scanning at 40× magnification and a field of view with the following measurements: horizontal—7.6 mm, vertical—5.7 mm. A test measurement was taken afterwards, creating a roughness line. Based on the ISO 21920-2:2023 standard [42], the short-wavelength lower limit *Ra* and the cut wavelength *λc* were set, depending on the obtained value. If Ra is more than 2 µm and less 10 µm, λs = 8 µm and λc = 2.5 mm. If *Ra* is more than 10 µm and less than 80 µm, λs = 25 µm and λc = 8 mm. Isometric views were created at 1200% magnification. Surface roughness measurements were taken three times for each sample. The direction of the surface roughness measurement profile with respect to the material deposition tracks is specified in Figure 3.

Further experimental testing was carried out on a DMG MORI CMX 600V vertical machining centre (DMG, Pleszew, Poland). The technical data of the machining centre are defined in Table 3.

**Figure 4:**
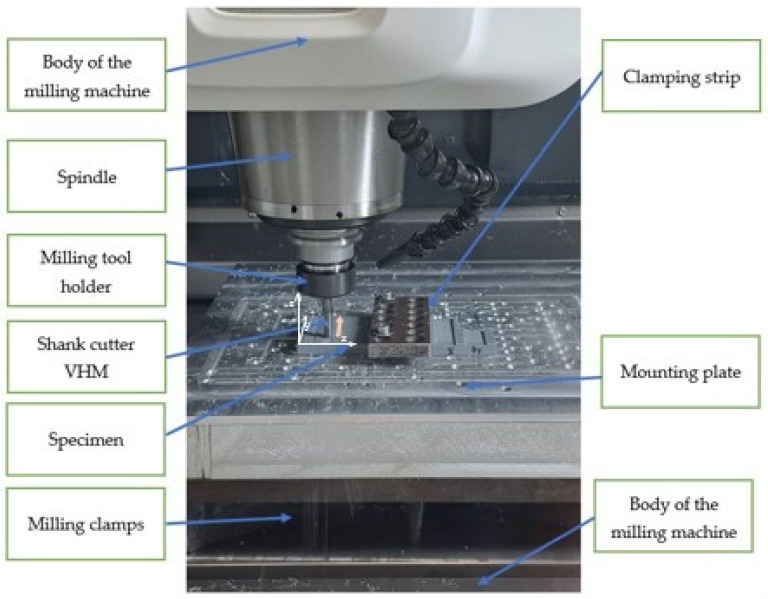
Workplace of DMG MORI CMX 600V.

The tests involved making straight grooves with an open geometry by milling a cuboid sample made using additive manufacturing from PETG material. The machining process was conducted without the use of cooling lubricants. The workpiece was placed on the table and fixed using a mounting plate. The fixing scheme is shown in Figure 4.

Three types of face milling cutters, made of different materials and with other geometric features, were used for machining. The cutters had a smooth cylindrical shank and were mounted in a HAIMER ISO 7388-1 SK40 collet chuck 40 (Haimer Gmbh, Igenhausen, Germany). Using a milling toolholder balanced to G2.5 25,000 rpm minimized the generation of centrifugal forces when the tool was rotated at high angular speed. This was important because it eliminated the occurrence of radial run-out and thus ensured stable working conditions during the cutting process as well as high surface quality. The milling toolholder was equipped with a tapered shank section with a taper of 7:24 made to DIN 69871 [49]. A clamping sleeve ER32D8 (APX Technologie Sp. z o.o., Opacz, Poland) was inside the holder, axially pressed against the conical surface of the holder socket using a locking nut. As a result of the sleeve being squeezed, the cutter became clamped.

The criteria for the selection of tools for testing referred to cutting ability—i.e., the ability of the tool to perform machining—and machinability, i.e., the susceptibility of the workpiece material to machining. The selected criteria were cutting edge durability, machined surface quality (roughness parameters and surface layer structure), and chip breakage. The material chosen for the study is PETG, which is easily machinable. The machining process is characterised by a low cutting temperature and a tendency to build up, resulting in poor machining quality. General recommendations for this polymer material are a cutting edge made of HSS, carbide, or PCD material with a polished rake surface.

Tool No. 1 is a carbide burr designed for machining polymer materials. It is equipped with a double-groove geometry to generate small chips, remove material efficiently, and consequently achieve a high surface quality. The tool is characterized by durability and wear resistance.

Tool No. 2 is a single-edge carbide burr. It is characterized by fast chip evacuation. During machining, it generates low cutting forces, reducing temperature in the cutting area. The mill is characterized by rigidity, wear resistance, and durability.

Tool No. 3 is a cylindrical face-milling cutter made of high-speed steel. It has a 30° chip groove angle to promote efficient chip removal. It is equipped with four blades. The tool is characterized by versatility, economy, and durability.

The tool specifications and cutting parameters used are shown below in Table 4.

**Figure 5:**
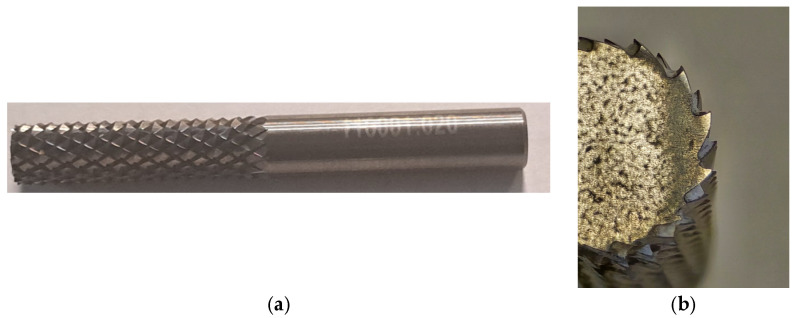
Tool No. 1: monolithic burr file cutter used in processing: (**a**) global view of burr file cutter and (**b**) partial view of burr file cutter.

**Figure 6:**
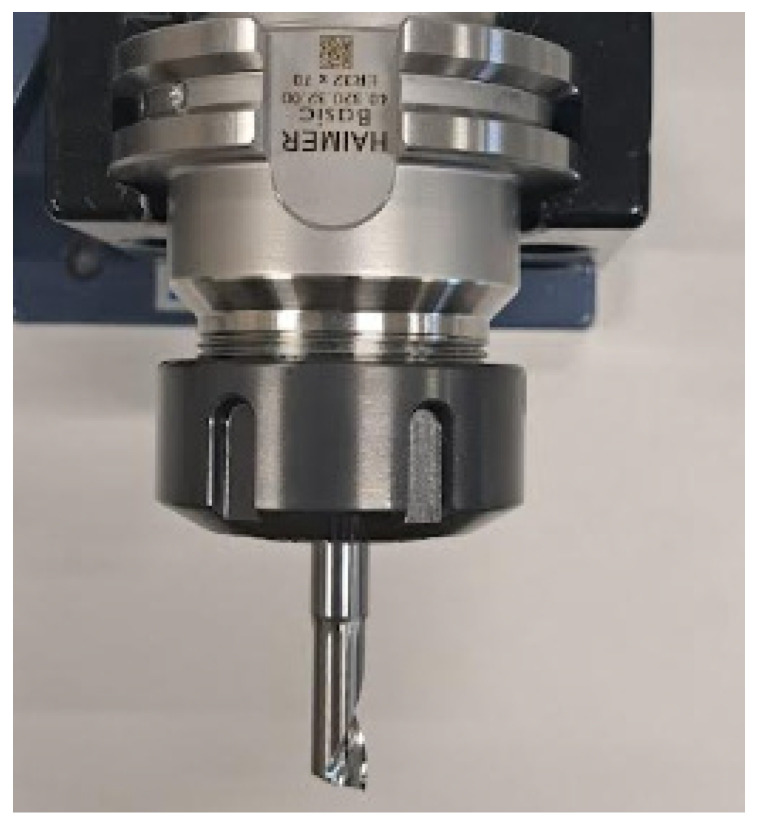
Tool No. 2: monolithic single-edge spiral burr cutter used in processing.

**Figure 7:**
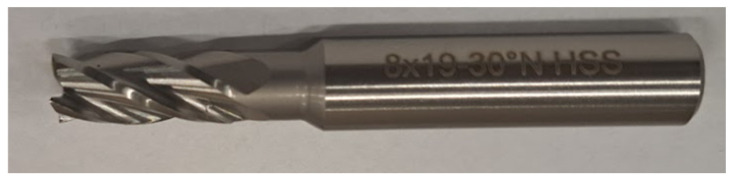
Tool No. 3: monolithic HSS spiral endmill straight shank used in processing.

The tools used for the milling operation are shown in Figure 5, Figure 6 and Figure 7.

Tool parameters such as length and diameter were measured with the machining tool in manual mode using a Haimer Microset UNO 20/40 pre-measurement and tool setting station. This device allows displacement in the *X* and *Z* axes within a range of 400 mm. The device features a grey cast-iron body, Heidenhain rulers, and Bosch Rexroth guides and bearings. The stand also has a telecentric IDS camera that magnifies the tool’s cutting edge 36 times. In addition, it has an edge finder function to automatically detect the blade’s shape.

**Figure 8:**
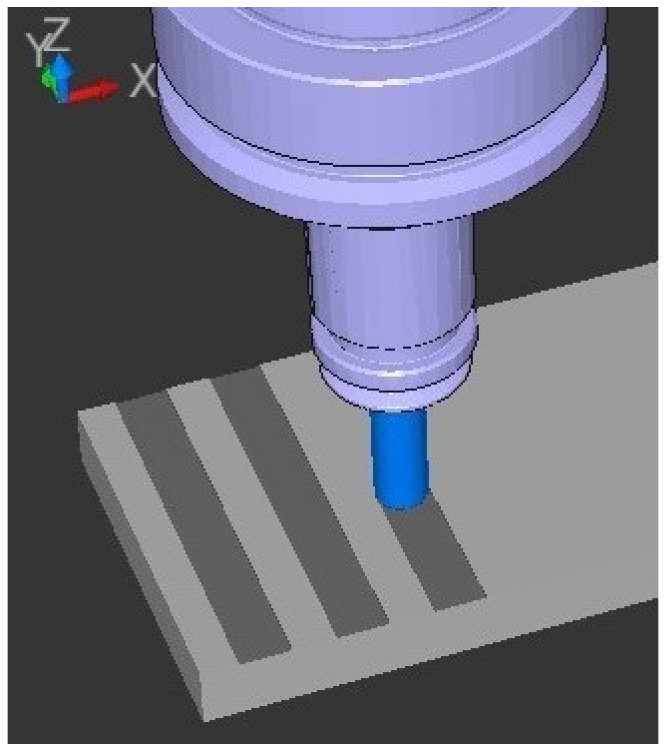
Manufacturing with NC code Heidenhain 640-based 3D simulation.

For comparative analysis, three grooves (complete tool diameter), 8 mm wide and 0.35 mm deep, were made on each sample (Figure 8). Each groove was machined with a different end mill, which allowed the influence of tool geometry and material on machining quality to be evaluated, depending on the printing and cutting parameters. The milling process was performed using a Heidenhain TNC 640 CNC postprocessor (DR. JOHANNES HEIDENHAIN Gmbh, Traunreut, Germany). Table 5 shows the numbering of the grooves produced and the corresponding 3D printing and cutting parameters used in the testing. The cutting parameters for the cutters were selected according to the literature or the manufacturer’s guidelines [50].

## 3. Results

The surface roughness values of the printed samples were measured before milling for layer heights of 0.1 mm and 0.2 mm. After the milling stage, further roughness measurements were taken for different printing layer heights *L_h_*, different feed rate during which the filament layer is extruded. Measurements were also carried out for two specific feed rates during milling *V_f_ =* 900 mm/min with a spindle speed of *n* = 6000 rpm and cutting depth *a_p_* of 0.35 mm and 400 mm/min with a spindle speed *n* of 1000 rpm.

**Figure 9:**
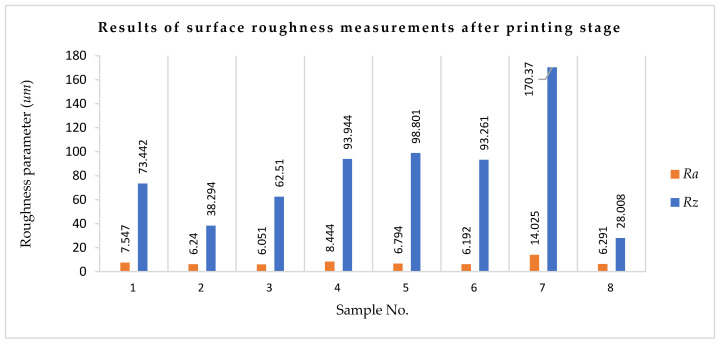
Results of surface roughness measurements after printing stage: *Ra*—roughness parameter and *Rz*—roughness parameter.

Table 6 and Figure 9 show results of surface roughness measurements on the rectangular sample after the printing stage.

**Figure 10:**
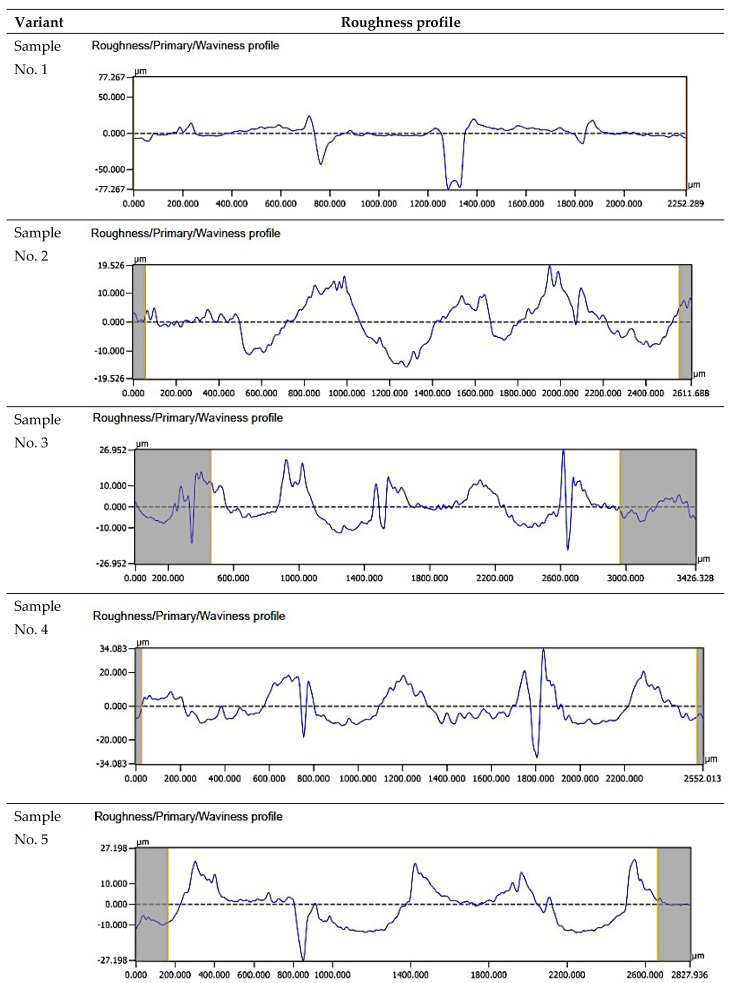

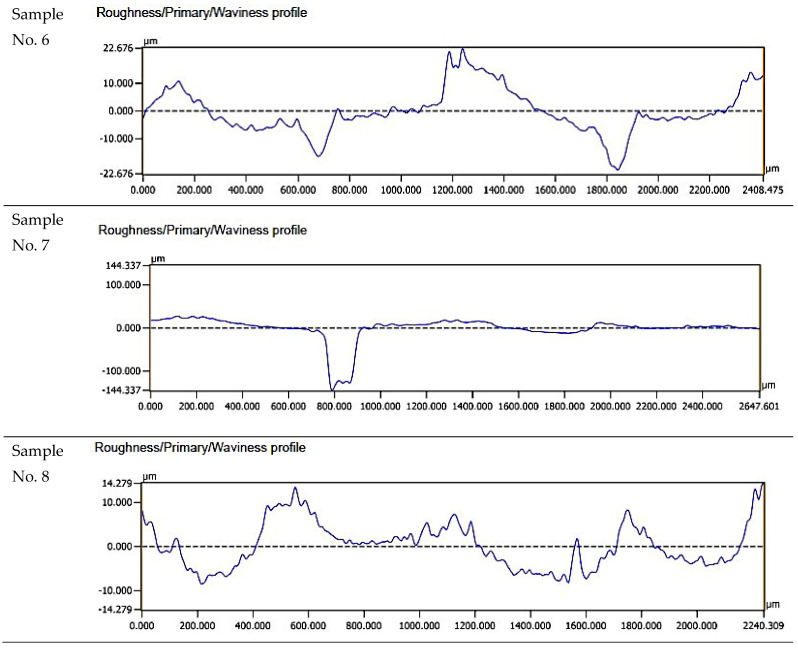
Comparison of the surface roughness profiles of the samples.

Figure 10 summarizes selected surface roughness profiles of the samples after the printing stage.

The surface roughness profiles after the printing stage are shown in Figure 9. The cut-off values are defined by ISO 4288 [51]. We set λs depending on the Ra (average roughness) of the target. If Ra was 2 µm or less, we set λs as 2.5 µm. If Ra was between 2 µm and 10 µm, we set λs as 8 µm. If Ra was between 10 and 80 µm, we set λs as 25 µm.

For sample No. 1, the roughness profile was recorded over a measurement section of 2252.289 µm. The maximum depression was 77.267 µm. The height distribution was irregular; in particular, the profile depressions dominated in the 1300–1400 µm range. For sample No. 2, the recorded roughness profile was taken over a measurement section of 2611.688 µm. The maximum elevation reached 19,526 µm, while the maximum depression was 17 µm. The distribution of elevations was irregular. For sample No. 3, the recorded roughness profile was taken from a measurement section measuring 2431.959 µm. The maximum elevation reached 34.083 µm, while the maximum depression was also 34.083 µm with respect to the average line. The distribution of elevations was irregular. For sample No. 4, the recorded roughness profile was taken over a measurement section of 2552.013 µm. The maximum elevation reached 12.917 µm, while the maximum depression was also 12.917 µm with respect to the mean line. The distribution of elevations was irregular. This was especially apparent in the range of 1700–1900 µm. For sample No. 5, the recorded roughness profile was taken from a measurement section of 2827.936 µm. The maximum elevation reached 25 µm, while the maximum depression was 27.198 µm with respect to the mean line. The distribution of elevations was irregular. For sample No. 6, the recorded roughness profile spanned a measurement section of 2408.475 µm. The maximum elevation reached 22.676 µm, and the maximum depression was also 22.676 µm relative to the average line. The distribution of elevations was irregular. For sample No. 7, the recorded roughness profile was over a measurement section of 2647.601 µm. The maximum elevation reached 10 µm, while the maximum depression was 144.337 µm with respect to the mean line. The elevation distribution was irregular, with depressions in the profile dominating in the 750–850 µm range in particular. For sample No. 8, the recorded roughness profile was taken over a measurement section of 2240.309 µm. The maximum elevation reached 14.279 µm, while the maximum depression was approximately 10 µm from the mean line. The elevation distribution was irregular.

**Figure 11:**
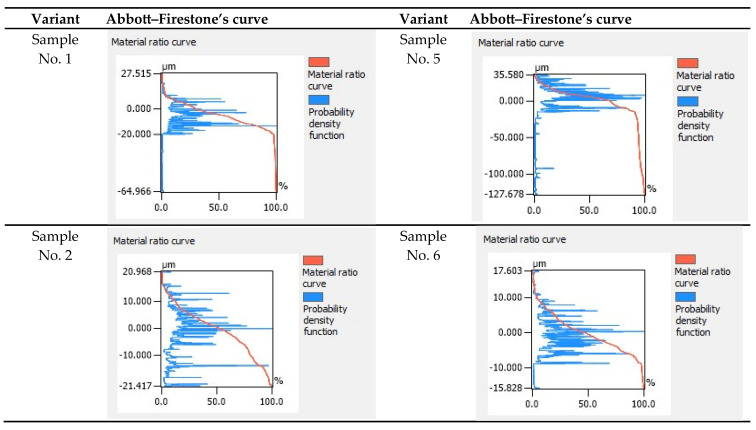

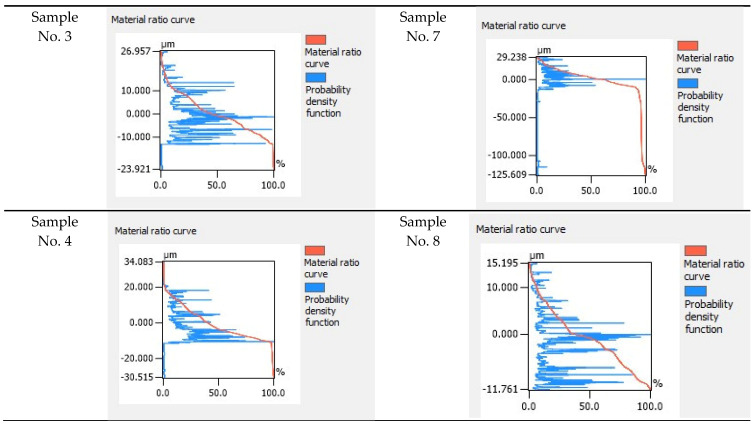
Abbott–Firestone curves for all 3D printing variants.

Figure 11 shows the Abbott–Firestone curves for the test samples after the printing stage. The shape of the individual curves varied depending on the printing parameters used. Based on Abbott–Firestone curves, we determined the percentage of material at a given height of the surface profile and the distribution of the height of points on the surface. The graph consists of two elements: the material share curve shown in red, and the probability density function shown in blue. The red line is the material ratio curve, while the blue line refers to the probability density function.

**Figure 12:**
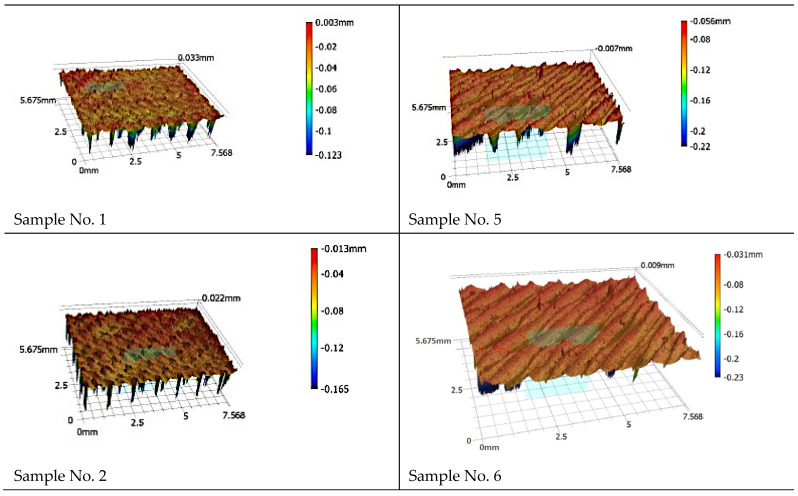

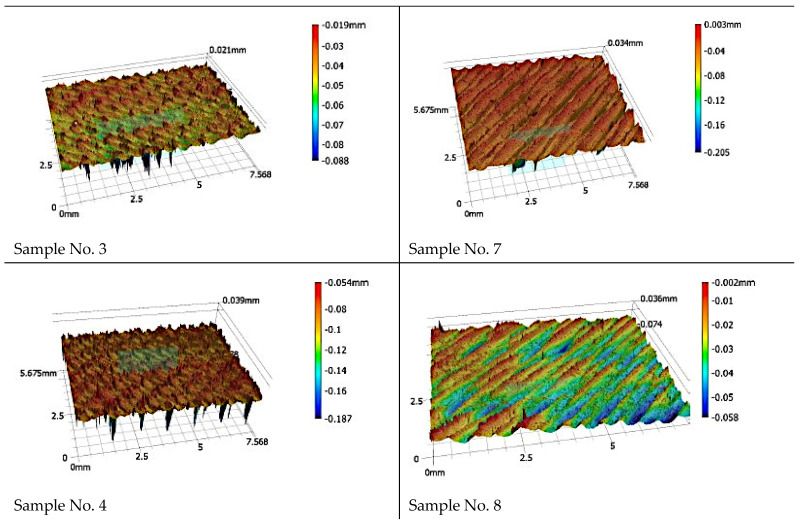
Isometric views of the surface after the 3D printing stage using different printing parameters.

Figure 12 shows the topographies of the surfaces created after the 3D printing stage using different printing parameters.

**Figure 13:**
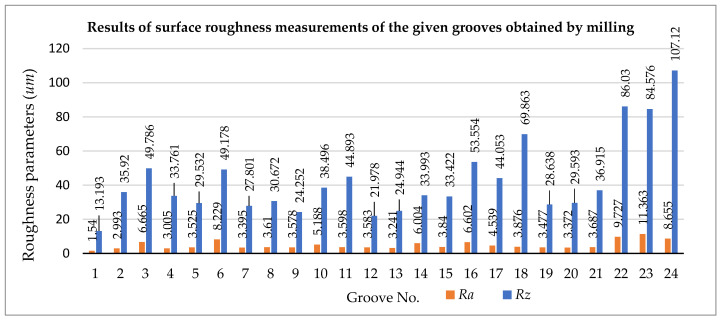
Results of surface roughness measurements of the grooves obtained by milling.

Table 7 and Figure 13 show selected 2D surface roughness parameters of the front surface of individual grooves of a rectangular cross-section made with burrs. The following 2D surface roughness parameters were analyzed: *Ra* (arithmetic mean of the ordinates of the roughness profile) and *Rz* (maximum height of the roughness profile). The standard deviation is given as a measure of the spread of the results obtained.

The results of the individual groove surface roughness measurements are summarized in ascending order in Table 8. The criterion used for compilation was the type of cutter used for the machining operation.

**Figure 14:**
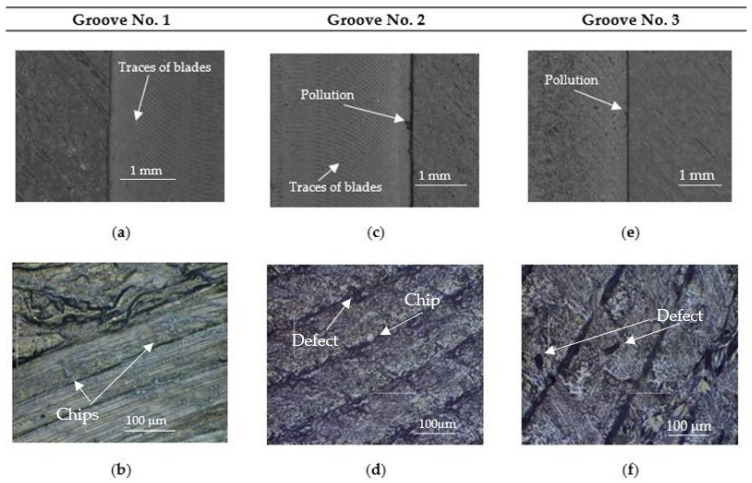
Surface microstructure of the rectangular sample after the milling stage: (**a**) Workpiece 1, groove 1, height of printing layer 0.1 mm, magnification × 40, *V_f_* = 900 mm/min, *n* = 6000 rpm, *a_p_* = 0.35 mm, tool No. 1: rotary burr. (**b**) Workpiece 1, groove 1, height of printing layer 0.1 mm, magnification × 600, *V_f_* = 900 mm/min, *n* = 6000 rpm, *a_p_* = 0.35 mm, tool No. 1: rotary burr. (**c**) Workpiece 1, groove 2, height of printing layer 0.1 mm, magnification × 40, *V_f_* = 900 mm/min, *n* = 6000 rpm, *a_p_* = 0.35 mm, tool No. 2: single-edge spiral burr cutter. (**d**) Workpiece 1, groove 2, height of printing layer 0.1 mm, magnification × 600, *V_f_* = 900 mm/min, *n* = 6000 rpm, *a_p_* = 0.35 mm, tool No. 2: single-edge spiral burr cutter. (**e**) Workpiece 1, groove 3, height of printing layer 0.1 mm, magnification × 40, *V_f_* = 400 mm/min, *n* = 1000 rpm, *a_p_* = 0.35 mm, tool No. 3: spiral endmill HSS. (**f**) Workpiece 1, groove 3, height of printing layer 0.1 mm, magnification × 600, *V_f_* = 400 mm/min, *n* = 1000 rpm, *a_p_* = 0.35 mm, tool No. 3: spiral endmill HSS.

Figure 14 summarizes the microstructures of the groove surfaces in sample No. 1 performed using 40× (Keyence VR-6000 profilometer) and 600× (Motic Inverted Microscope) magnification. Contamination was observed after processing, adhering to the edge of groove No. 2 and groove No. 3. The surface shows traces of the blades and their regular occurrence. Some dependence of the surface morphology on the type of milling tool used could be observed. When the milling operation was performed using tool No. 1, resulted in a groove surface characterized by regular blade marks, uniform path spacing, an absence of burrs, and good surface quality. In contrast, tool No. 2 produced irregularities within the groove in the form of fragments of material stuck to the edges and morphological defects (voids) in the structure. Conversely, the groove made with tool No. 3 had no visible blade marks, but the surface was delaminated and showed numerous structural defects.

## 4. Discussion

Tool No. 1 (Figure 5) is a multi-blade monolithic file shank cutter, which uses different inclinations of the chip grooves. The cutter has no leading cutting edges. After treatment with this tool, the highest surface roughness was obtained for groove No. 22, where the following parameters were assumed for the 3D printing process: print layer height *L_h_* = 0.2 mm and *V_feed_* = 120 mm/s. The lowest surface roughness value was obtained for groove No. 1; the roughness parameter was *Ra* = 1.54 μm.

Another tool used to make the through-groove was a monolithic single-point cutter (tool No. 2, Figure 6). The cutter has one cutting spiral with a positive geometry. The chips are discharged upwards (towards the spindle), which is beneficial as material particles do not accumulate in the chip groove. The lowest surface roughness after the machining stage with this tool at *Ra* = 2.993 μm was obtained for sample number 1, groove No. 2 for which the feed rate was *V_feed_* = 120 mm/s during 3D printing, and the thickness of the applied layer *L_h_* was 0.1 mm. The other settings were as follows: spindle rev. *n* = 6000 rpm; feed rate during milling *V_f_* = 900 mm/min; and depth of cut *a_p_* = 0.35 mm. Meanwhile, the highest surface roughness value after cutting was observed for sample number 8, where the roughness value of groove number 23 was *Ra* = 11.363 μm, for which the feed rate during 3D printing was *V_feed_* = 120 mm/s, and the thickness of the applied layer *L_h_* was 0.2 mm.

Tool No. 3 (Figure 7) is a monolithic cylindrical-face four-blade burr with a countersink on the face made from standard high-speed steel. The middle part of the tool is not actively involved in the cutting process. The cutting edge inclination angle *λ_S_* was 30°, and the cutting edge angle *κ_r_* was 3°. The smallest surface roughness at *Ra* = 3.578 μm was obtained for sample number 3, for which the feed rate during 3D printing was *V_feed_* = 100 mm/s and the thickness of the applied layer *L_h_* was 0.1 mm. The other settings were as follows: spindle rev. *n* = 1000 rpm; feed rate during milling *V_f_* = 400 mm/min; and cutting depth *a_p_* = 0.35 mm. Meanwhile, the highest surface roughness value after cutting was observed for sample number 8, where the roughness value of groove number 24 was *Ra* = 8.655 μm, for which the feed rate during 3D printing was *V_feed_* = 120 mm/s, and the thickness of the applied layer *L_h_* was 0.2 mm.

The lowest surface roughness after *Ra* = 1.54 μm was obtained for sample number 1, groove No. 1. The 3D printing process used a feed rate of *V_feed_* = 120 mm/s and layer thickness *L_h_* = 0.1 mm. Finishing was performed using milling with a spindle speed *n* of 6000 rpm, feed rate *V_f_* = 900 mm/min, and depth of cut *a_p_* = 0.35 mm. The No. 1 tool used is a rotary burr dedicated to machining composite materials.

In contrast, the highest surface roughness after the machining stage was observed for sample number 8, where the roughness value of groove number 23 was *Ra* = 11.363 μm. The sample was produced using 3D printing with a feed rate of *V_feed_* = 120 mm/s and a layer thickness of *L_h_* = 0.2 mm. The milling process used tool No. 2, the single-edge spiral burr cutter.

Analysis of the Abbott curves of the printed samples provided information on the random distribution of elevations and depressions. The maximum height of the measured profile was 35.580 μm for sample No. 5. The minimum depth of the measured profile was −125.609 μm for sample No. 7. The parameters describing the bearing capacity indicated that for the analytical area of the test samples (No. 1, No. 3, No. 4, and No. 6), the load-bearing shares of the vertices and pits were approximately constant at 5% and 95%, respectively. The shape of the curve also remained unchanged, with an S-shaped curve typical of surfaces with randomly distributed irregularities. For samples No. 1 and No. 6 and groove No. 8, the distribution of surface profile heights was asymmetrical, with the highest density of data in the range of negative values. The highest density of positives was in sample No. 5. The height range of the roughness profile for sample No. 7, ranging from 125.609 to 29.238 μm, indicated inaccurate deposition of the filament on the layer, resulting in surface irregularities.

Figure 10 illustrates three-dimensional maps of the surface topography of the specimens prepared for additive testing. The choice of this technology is evident in the images, as the surface structure is characterized by clearly defined bands, corresponding to successive paths of the applied filament. When analyzing the colour scale, it was noted that the profile height ranges from −0.23 mm (blue, sample No. 6) to 0.003 mm (red, sample No. 1). Local elevations and depressions were visible on the surface of the samples; their presence on the surface indicates the occurrence of printing defects.

## 5. Conclusions

This study aimed to improve the surface quality of the grooved specimens by using hybrid machining, i.e., a combination of additive manufacturing technology (FDM) and milling. Three cutting tools were examined, which were burrs that differed in blade geometry and material of manufacture but had the same diameter, i.e., 8 mm. The two grooves’ cutting parameters were set to *n* = 6000 rpm and *V_f_* = 900 mm/min. This was because they are cutters specifically for machining composite materials.

Test samples printed at 100% fill, 225 °C extruder temperature, and 70 °C table temperature showed no signs of distortion.

The developed hybrid technology made it possible to obtain a component surface roughness parameter *R_a_* equal to 1.54 μm for a filament layer height *L_h_* = 0.1 mm, feed rate *V_feed_* = 120 mm/s, and layer height *L_h_* = 0.2 mm, with *V_feed_* = 90 mm/s and *R_a_* being 3.241 μm.

Interpreting the results obtained, it can be concluded that the use of hybrid machining translates into lower values of roughness parameters. The implementation of a lower layer height during printing and the use of a multi-blade cutter to create a through-groove provide better surface roughness.

Comparing the surface microstructure of the element to be printed with layer heights defined as 0.1 mm and 0.2 mm, it can be noted that better bonding of the filament layers occurred with the samples for which a 0.1 mm height was used. Greater adhesion between the layers also translated into a more accurate representation of the model geometry and a lower surface roughness parameter.

The relationship developed is for a specific filament and given environmental conditions. However, the given relationship can be used to predict the roughness parameter for filaments with similar characteristics.

## Figures and Tables

**Table 1:** Three-dimensional printing parameters used in printing workpieces.

Parameter	Value
Filament	PETG
Extruder temperature is maintained during printer operation, *E_t_*	225 °C
Temperature of the platform for all extruded filament layers, *P_t_*	70 °C
Infill density of the sample, *I_d_*	100%
Number of outer layers of the printed object, *n*	4
Solid bottom layers	4
Solid top layers	4
Filament layer height, *L_h_*	0.1 and 0.2 mm
Height of the first layer, *L_h1_*	0.25 mm
Width of the first layer, *W_h_*	0.4 mm
Infill pattern inside the sample, *I_p_*	linear
Feed rate during which the filament layer is extruded, *V_feed_*	90 mm/s; 100 mm/s; 110 mm/s; 120 mm/s

**Table 2:** Technical data of the Keyence VR-6000 3D profilometer.

Head		VR-6100							
Camera		Low magnification (wide field of view)				High magnification (high resolution)			
Magnification		12×	25×	38×	50×	40×	80×	120×	160×
Field of view	Horizontal (mm)	24	12	8	6	7.6	3.8	2.5	1.9
	Vertical (mm)	18	9	6	4.5	5.7	2.9	1.9	1.4
Height measurement		Display resolution 0.1 µm							
Height measurement range		50 mm				30 mm			
XY measurable range		92 × 86 mm							

**Table 3:** Technical data of the CMX 600V vertical machining centre.

MODEL	CMX 600V
Max spindle speed (rpm)	12,000
Power supply (V)	230
Max. workpiece height (mm)	630
Max. workpiece length (mm)	900
Max. workpiece width (mm)	560
Max. workpiece weight (kg)	600
Working surface (mm)	900 × 560
Mass of machine (kg)	5000
Total power requirement (kVA)	32

**Table 4:** Tool specifications and cutting parameters used.

Type of Cutter	Tool Designation	Size	Number of Blades	Manufacturer	Recommendation	Machining Parameters
Tool No. 1—Rotary burr (Figure 4)						
Carbon roughing end mill	116001.020	8.0 × 25 × 8 × 63	Data not available	Karnasch Professional Tools, Mannhei, Germany	VHM milling cutter for composites: Carbon fibre-reinforced plastics CFK, CFRP; Glass fibre-reinforced plastics GFK, GFRP; Aluminium CFK and aluminium CFRP, Honeycomb composites, CFK, CFK, CFRP, GRP.	*n* = 6000 rpm *V_f_* = 1000 mm/min *a_p_* = 0.35 mm *f_z_* = 0.04 mm/tooth
Tool No. 2—Single-edge spiral burr cutter (Figure 5)						
VHM helical cutter for plastics	Solid Carbide (VHM)	8.0 × 27 × 60	*z* = 1	STB Tools, Swarzędz, Poland	VHM milling cutter for plastics, aluminium and its alloys, copper, and other light metals.	*n* = 6000 rpm *V_f_* = 1000 mm/min *a_p_* = 0.35 mm *f_z_* = 0.16 mm/tooth
Tool No. 3—Spiral endmill (Figure 6)						
Spiral endmill for metal	NFPa 0641-512-100-080 HSS	8.0 × 19 × 10 × 69	*z* = 4	FENES S.A., Siedlce, Poland	HSS spiral endmill for finishing and semi-finishing work on metals, stainless steel, cast-iron, aluminium alloys, copper, and other light metals.	*n* = 1000 rpm *V_f_* = 400 mm/min a_p_ = 0.35 mm *f_z_* = 0.1 mm/tooth

**Table 5:** Groove numbering and the printing and cutting parameters used. *L_h_*—filament layer height, *V_feed_*—feed rate during which the filament layer is extruded, *n*—the rotational speed of the cutting tool that carries out the main movement, and *V_f_*—feed rate.

Sample No.	Groove No.	*L_h_* (mm)	*V_feed_* (mm/s)	Tool No.	*n* (rpm)	*V_f_* (mm/min)
1	1	0.1	120	1	6000	900
1	2	0.1	120	2	6000	900
1	3	0.1	120	3	1000	400
2	4	0.1	90	1	6000	900
2	5	0.1	90	2	6000	900
2	6	0.1	90	3	1000	400
3	7	0.1	100	1	6000	900
3	8	0.1	100	2	6000	900
3	9	0.1	100	3	1000	400
4	10	0.1	110	1	6000	900
4	11	0.1	110	2	6000	900
4	12	0.1	110	3	1000	400
5	13	0.2	90	1	6000	900
5	14	0.2	90	2	6000	900
5	15	0.2	90	3	1000	400
6	16	0.2	100	1	6000	900
6	17	0.2	100	2	6000	900
6	18	0.2	100	3	1000	400
7	19	0.2	110	1	6000	900
7	20	0.2	110	2	6000	900
7	21	0.2	110	3	1000	400
8	22	0.2	120	1	6000	900
8	23	0.2	120	2	6000	900
8	24	0.2	120	3	1000	400

**Table 6:** Results of surface roughness measurements on the rectangular sample after the printing stage: *Ra*—roughness parameter, *Rz*—roughness parameter, *L_h_*—printing layer height, $\bar{X}$—arithmetic mean of three measurements, σ—standard deviation.

Sample No.	*Ra* (μm)		*Rz* (μm)		*L_h_* (mm)	*V_feed_* (mm/s)
	$\bar{X}$	**σ**	$\bar{X}$	**σ**		
1	7.547	1.300	73.442	40.802	0.1	120
2	6.240	0.815	38.294	3.731	0.1	90
3	6.051	1.321	62.510	47.422	0.1	100
4	8.444	1.042	93.944	79.600	0.1	110
5	6.794	0.699	98.801	62.835	0.2	90
6	6.192	0.620	93.261	66.700	0.2	100
7	14.025	1.047	170.37	14.893	0.2	110
8	6.291	0.342	28.008	4.533	0.2	120

**Table 7:** Results of surface roughness measurements on the rectangular sample of the given grooves obtained by milling: *Ra*—roughness parameter, *Rz*—roughness parameter, $\bar{X}$—arithmetic mean of three measurements, and σ—standard deviation.

Sample No.	Groove No.	*Ra* (μm)		*Rz* (μm)	
		$\bar{X}$	**σ**	$\bar{X}$	**σ**
1	1	1.54	0.157	13.193	2.396
	2	2.993	0.565	35.920	9.487
	3	6.665	1.631	49.786	14.877
2	4	3.005	0.774	33.761	16.192
	5	3.525	0.650	29.532	8.407
	6	8.229	1.159	49.178	4.425
3	7	3.395	0.709	27.801	9.792
	8	3.610	0.628	30.672	6.449
	9	3.578	0.537	24.252	5.194
4	10	5.188	1.811	38.496	8.396
	11	3.598	0.590	44.893	22.319
	12	3.583	0.661	21.978	5.475
5	13	3.241	0.524	24.944	6.647
	14	6.004	0.663	33.993	4.285
	15	3.840	0.994	33.422	14.278
6	16	6.602	0.540	53.554	8.001
	17	4.539	0.557	44.053	12.837
	18	3.876	2.924	69.863	41.873
7	19	3.477	1.050	28.638	6.713
	20	3.372	0.575	29.593	8.090
	21	3.687	0.418	36.915	9.757
8	22	9.727	1.212	86.030	8.190
	23	11.363	3.984	84.576	28.040
	24	8.655	2.307	107.120	19.957

**Table 8:** Presentation of the results of the roughness measurements of the given grooves obtained by milling with the individual milling tools in ascending order.

*R_a_*, (μm)	1.54	3.005	3.241	3.395	3.477	5.188	6.602	9.727
Sample No.	1	2	5	3	7	4	6	8
Lh, (mm)	0.1	0.1	0.2	0.1	0.2	0.1	0.2	0.2
Vfeed, (mm/s)	120	90	90	100	110	110	100	120
n, (rpm)	6000	6000	6000	6000	6000	6000	6000	6000
Vf, (mm/min)	900	900	900	900	900	900	900	900
Tool No. 1	Rotary burr							
*R_a_*, (μm)	2.993	3.372	3.525	3.598	3.61	4.539	6.004	11.363
Sample No.	1	7	2	4	3	6	5	8
Lh, (mm)	0.1	0.2	0.1	0.1	0.1	0.2	0.2	0.2
Vfeed, (mm/s)	120	110	90	110	100	100	90	120
n, (rpm)	6000	6000	6000	6000	6000	6000	6000	6000
Vf, (mm/min)	900	900	900	900	900	900	900	900
Tool No. 2	Single-edge spiral burr cutter							
*R_a_*, (μm)	3.578	3.583	3.687	3.84	3.876	6.665	8.229	8.655
Sample No.	3	4	7	5	6	1	2	8
Lh, (mm)	0.1	0.1	0.2	0.2	0.2	0.1	0.1	0.2
Vfeed, (mm/s)	100	110	110	90	100	120	90	120
n, (rpm)	1000	1000	1000	1000	1000	1000	1000	1000
Vf, (mm/min)	400	400	400	400	400	400	400	400
Tool No. 3	Spiral endmill							

## Data Availability

The data presented in this study are available on request from the corresponding author due to privacy restrictions.
